# Cell Therapy Approaches for Articular Cartilage Regeneration

**DOI:** 10.1080/15476278.2023.2278235

**Published:** 2023-11-14

**Authors:** Meagan J. Makarczyk

**Affiliations:** a Department of Bioengineering, University of Pittsburgh, Pittsburgh, Pennsylvania, USA; b Department of Orthopaedic Surgery, University of Pittsburgh, Pittsburgh, Pennsylvania, USA

**Keywords:** Cartilage, stem cell, synthetic biology

## Abstract

Articular cartilage is a common cartilage type found in a multitude of joints throughout the human body. However, cartilage is limited in its regenerative capacity. A range of methods have been employed to aid adults under the age of 45 with cartilage defects, but other cartilage pathologies such as osteoarthritis are limited to non-steroidal anti-inflammatory drugs and total joint arthroplasty. Cell therapies and synthetic biology can be utilized to assist not only cartilage defects but have the potential as a therapeutic approach for osteoarthritis as well. In this review, we will cover current cell therapy approaches for cartilage defect regeneration with a focus on autologous chondrocyte implantation and matrix autologous chondrocyte implantation. We will then discuss the potential of stem cells for cartilage repair in osteoarthritis and the use of synthetic biology to genetically engineer cells to promote cartilage regeneration and potentially reverse osteoarthritis.

## Introduction

Articular cartilage (AC) is a specialized cartilage found in many joints of the body such as the knees and hips. Particularly, in the knee joint, the cartilage functions to facilitate and support the movement between the tibia and the femur and distributes force during mechanical load.^1^ Avascular and anural in nature, the AC is composed primarily of water and extracellular matrix (ECM) proteins secreted by chondrocytes, the primary cell type of the cartilage.^1,2^ Chondrocytes produce ECM proteins, most abundantly aggrecan and collagen type II along with other proteoglycans.^3,4^ The cartilage ECM is essential for proper cartilage functionality and biomechanical properties, yet chondrocytes are limited in their regenerative capacity.^4^ Furthermore, there are multiple tissue components of the knee joint that assist with cartilage maintenance, such as the synovium and infrapatellar fat pad.^3^ The synovium plays an invaluable role by providing nutrients and lubricating the cartilage,^5^ and the infrapatellar fat pad supports and cushions the cartilage during load.^6^ Collectively, the bone, cartilage, fat pad, and synovium make up the knee-joint organ.

Cartilage pathologies can be mentally and physically debilitating and can lead to additional economic burden.^3^ To date, there are not any FDA approved disease modifying drugs to treat cartilage pathologies such as osteoarthritis (OA), which is the 11^th^ global contributor to disability.^7,8^ Knee cartilage defects also known as cartilage lesions are common^9, 10^ and can occur due to aging, obesity, mechanical injury, and gender.^3^ Cartilage lesions are associated with pain and loss of full mechanical function,^10^ and untreated cartilage defects have the potential to result in OA.^11,12^ Cell therapies are primarily used for cartilage defects such as autologous chondrocyte implantation (ACI) and matrix autologous chondrocyte implantation (MACI) which have been effective but are limited to patients under the age of 45. 38–47% of adults in the U.S. over the age of 60 are predicted to have OA.^11^ Therefore, the treatment of cartilage defects prior to the onset of OA is vital. The current gold standard treatment for OA is total joint arthroplasty, but this is also not suitable to all patients.^3,13^ In this review, we will discuss the current cell therapies for cartilage defects and OA, the potential of stem cells for cartilage regeneration, and finally, with a look at future perspectives of synthetic biology approaches to cartilage engineering.

## Cell therapies for cartilage regeneration

### Microfracture and grafting: the current gold standards

**Figure 1.**
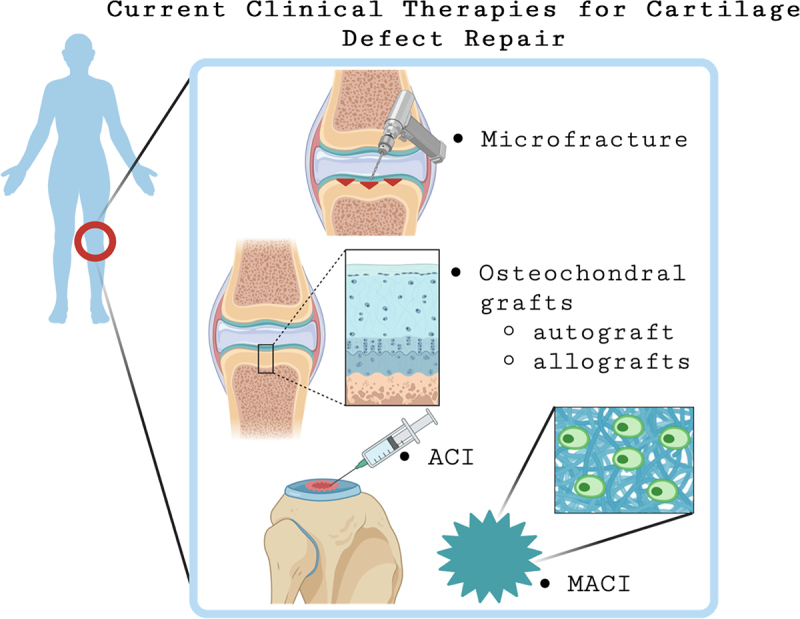
Schematic summary of therapies for cartilage defect regeneration that are FDA approved and practiced in the clinic. Treatments like microfracture and autografts are the current gold standard for cartilage defect repair. ACI and MACI have reached FDA approval and can be used for large defect repair.

Microfracture (MF) also known as marrow stimulation, is used for cartilage defects less than 2 cm^2^. During this procedure, small portions of the subchondral bone are penetrated allowing for a conduit between the bone marrow and the cartilage. Small holes are drilled into the bone beneath the defective cartilage, allowing for bone marrow stimulation and is depicted in Figure 1.^14^ Stem cells in the bone marrow assist with cartilage regeneration and healing of the defect area.^12, 15, 16^ However, MF focuses more on the repair of fibrocartilage rather than the actual regeneration of the hyaline cartilage.^17,18^ Patients that received MF have shown a decline in joint functionality after 24 months of surgery. There is also still a concern that MF does not delay the eventual onset of OA.^14^ Overall, the quality and repair of the cartilage following MF is variable and inconsistent,^18^ which is thought to be linked to the limited low number of stem cells that invade the damaged area following stimulation. The lack of standardization and clinical follow ups for patients makes it difficult to track the success rate of MF repair and compare different clinical outcomes.^18^

Other gold standard treatments involve the use of osteochondral grafts to repair larger (>2 cm^2^) lesions.^19,20^ Autografts are pieces of osteochondral tissue removed from a lesser loading bearing portion of the joint and transferred into the lesion.^15^ By physically filling the patients defect with healthy tissue, the hope is that the tissue will integrate into the damaged site, thus repairing the defect. Extensive clinical studies have demonstrated the efficacy of osteochondral autografts for defect repair following 10 years post operation, and a benefit of this procedure was reported to be rapid graft integration at the defect site.^15^ Osteochondral autografts have demonstrated their own challenges such as limited tissue availability and donor site morbidity. Physiologically, the area of the knee joint associated with the lowest mechanical pressure is located at the lateral femoral trochlea or femoral notch and is the site where most plugs are derived. Based on plug size, contact pressures in the knee can contribute to donor site morbidity and pain, thus contributing to further complications for the patient.^21^ To mitigate donor site morbidity, an alternative technique known as mosaicplasty has been used, in which multiple, small plugs are harvested from a portion of heathy cartilage tissue and placed into small to medium-sized defect sites.^22^ The use of the patient’s own tissue and further damage to the knee joint is not ideal and further necessitates alternative strategies for defect repair. Figure 1 depicts the current treatment methods for cartilage defect repair.

**Figure 2.**
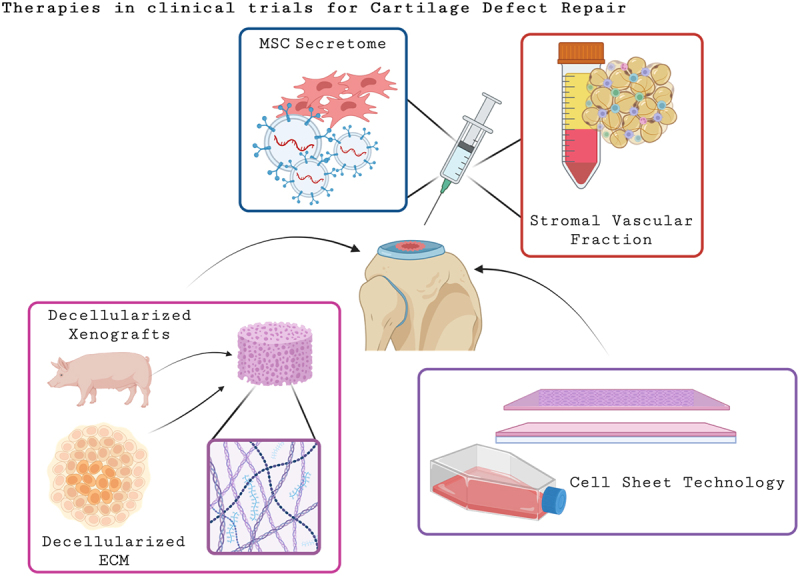
Schematic summary of therapies currently in clinical trials for cartilage defect regeneration. Implanted therapies such as decellularized scaffolds and cell sheet technology are being used to promote cartilage regeneration and fill defects. Additionally, the MSC secretome and stromal vascular fraction from adipose tissue have been intra-articularly injected into the knee with the hope that the secretome from these cells will result in a therapuetic effect. A combination of the therapeutics such as decellularized ECM paired with the stromal vascular faction has also been investigated.

Other alternatives to autografts is osteochondral allografts which are commonly harvested from young donors within 24 h after death, but have a short shelf-life of 28 days and immunogenic concerns.^20^ Nonetheless, while these treatments have demonstrated clinical success, the feild of tissue engineering and regenerative medicine is to engineer approaches for cartilage regeneration to overcome these limitations. Within the past two decades, more research has been conducted on the use of xenografts for osteochondral defect repair. Here in, osteochondral plugs, from animals such as porcine knees are decellularized and have shown efficacy in preclinical and clinical studies as shown in Figure 2.^19^

The use of decellularized ECM has become more common in which it has been suggested that using the architecture of the native cartilage ECM has the potential to promote hyaline-like cartilage regeneration.^23–25^ The primary concern regarding xenotransplantation involves immunogenic rejection of the donor tissue. However, antigens that cause immune response in humans can be removed via the decellularization process. For example, the α-gal epitope is found on all cell surface components of glycoproteins and glycolipids of all mammals and is the primary contributor to transplant rejection and immune response in humans. Protocols removing the α-gal epitope have been standardized through the decellularization process.^19, 20, 26^ Therefore, the use of decellularized ECM for cartilage repair has become increasingly investigated.

Decellularization of porcine osteochondral xenografts for cartilage regeneration has reached preclinical and clinical studies. A study conducted by Kheir et al. demonstrated the removal of cells and the α-gal epitope in porcine xenografts that were then implemented in an *in vivo* mouse model.^19^ A different study from Adib et al. used decellularized osteochondral grafts from sheep femurs and combined it with the following biological components: platelet-rich fibrin, amniotic membrane extract, or rabbit bone marrow-derived mesenchymal stromal cells. The ECM and biological combination groups were then used to treat osteochondral defects in rabbits. The regeneration of cartilage and bone in the ECM and amniotic membrane extract was determined to be the most efficacious.^27^ As demonstrated in this study, biologics can be combined with decellularized scaffolds to promote hyaline cartilage regeneration.

More recent studies have used differentiated stem cells to form the cartilage ECM, decellularized it and integrated native chondrocytes into the decellularized scaffold. It has been noted that there is a pro-chondrogenic effect of decellularized matrices linked to glycogen synthase kinase-3 beta (GSK3β) pathway which is crucial for the regulation of cell differentiation.^28^ Moreover, this study demonstrated the use of induced pluripotent stem cell (iPSC)-derived chondrocyte decellularized ECM as a potential matrix for cartilage regeneration in chondral defects. Mechanistic analysis linked the pro-chondrogenic effects of the decellularized matrix to the GSK3β- wingless-related integration site (WNT) signaling pathway.^28^ Decellularized ECM, regardless of the source has become a more prevalent natural matrix used for cartilage tissue engineering and shows great potential as a cell-free therapeutic and is also depicted in Figure 2. In the next section, we will focus on scaffold-free and matrix chondrocyte-based therapies for cartilage repair.

### Chondrocyte therapies: a focus on autologous chondrocyte implantation

For the past two decades, more research has involved the injection of autologous chondrocytes to promote cartilage regeneration in patient defects. This is known as autologous chondrocyte implantation (ACI).^29,30^ ACI is a two-step method for addressing cartilage defects, typically larger than 2 cm^2^. The first step of this procedure is the isolation of chondrocytes from a specimen of healthy cartilage in a non-load bearing portion of the knee.^15^ These chondrocytes are then expanded in monolayer and injected back into the patient’s defect. The first generation of this technique used a periosteal patch to isolate the chondrocyte suspension to the defect area.^15, 29, 31^ However, issues with chondrocyte hypertrophy, high re-operation and debridement rates necessitated the second generation of ACI.^32^

This new generation saw a shift in patch material from periosteal patch to a collagen type I and III porcine membrane.^31,32^ This method still required membrane suturing to the defect and resulted in some suspension leaking but it did prove to be more effective.^15^ The tissue maturation following ACI has been categorized into four stages: implantation state (0–6 weeks after implantation), transition and proliferation stage (6–12 weeks), early maturation stage (12–26 weeks), and the late maturation stage (26 weeks- 3 years). During the implantation stage, the chondrocytes begin to migrate into the defect and then fill the defect and create soft cartilage in the transition and proliferation stage. Early maturation is considered the point at which chondrocytes begin to produce collage type II and aggrecan shifting from a primitive cartilage to a more solid cartilage. Lastly, during the late maturation stage, the new tissue has filled the defect area and cartilage reaches a hyaline-like status.^31^

Despite some limitations of the device, ACI is an effective treatment method for cartilage lesions and poses benefits such as a lack of immunogenicity through the use of the patient’s own cells.^29^ Chondrocyte leaking and overall integration into the cartilage prompted further improvement. The third generation of the ACI technique incorporated a matrix scaffold known as Matrix-Assisted Autologous Chondrocyte Implant (MACI)^15, 31^ to bolster chondrocyte viability and mimic the mechanical properties of the cartilage.^15,30^ More details regarding MACI will be discussed in the next section.

### Chondrocyte therapies: matrix-assisted autologous chondrocyte implantation

MACI is one of the current and most common cell therapy approaches for cartilage defects which was approved by the FDA in 2016. Chondrocytes are isolated from patient cartilage, expanded, then seeded on a 3D matrix composed of a porcine collagen membrane.^33,34^ This established medical device incorporates both a matrix and cells for enhanced cartilage regeneration to fill cartilage defects. As chondrocytes are limited in availability throughout the knee the idea behind this device is to promote the integration of chondrocytes back into the native cartilage, thus filling the defect. MACI is applicable for a range of cartilage defects from single to multiple symptomatic defects.^35^ Common chondral defects in the knee include: lateral femoral condyle, medial femoral condyle with and without damage to the bone, patellar, and trochlea defects.^35^

As mentioned previously, the most abundant ECM protein of the cartilage is type II collagen, however MACI is composed of type I/III collagen.^14,29^ The matrix portion of MACI is dual sided for multi-faceted integration into the knee; one side is equipped for cell adhesion and proliferation while the other functions to decrease friction within the knee joint.^10^ To secure the matrix to the native cartilage a fibrin sealant is used. Similar to ACI, isolation of chondrocytes for MACI requires surgical intervention to extract a piece of healthy cartilage in which autologous chondrocytes are collected and expanded in monolayer.^36^ Once chondrocytes have been expanded, they are uniformly seeded using a uniform loading unit (ULU™) onto the porcine collagen membrane and cultured. The desired cell density on the matrix is 500,000 to 1,000,000 cells per cm.^2^ When the desired cell density is reached MACI is sent to the location of surgery^36^ where a trained surgeon performs a mini-arthrotomy and debrides the cartilage defect. The matrix is then cut to fit the shape of the defect and implanted into the knee with the cell seeded side facing the subchondral defect and the reduced friction side facing the joint exterior.^29,36^ The autologous aspect of this study limits adverse immune response due to the use of the patient’s own cells and avoids concerns posed by stem cells, such as cost, immune response, and tumorgenicity. However, the need to biopsy healthy cartilage from patients for the isolation of chondrocytes limits this device. The integration of stem cells into this model could provide an unlimited cell source for cartilage regeneration, and no longer necessitate the additional surgery to isolate autologous chondrocytes. Additionally, MACI is limited to cartilage lesions and defects, and it is contraindicated for the use in patients with severe or inflammatory OA and under the age of 18 and over the age of 55.^36^ The target population for cartilage treatment are people over the age of 60. A range of 38–47% of adults over the age of 60 are predicted to have OA in the U.S., and that number is expected to rise with increase of the aging population.^11^

### Chondrocyte therapies: cell sheets

Advances in tissue engineering have modified approaches like MACI and new methods such as cell sheet technologies. Cell sheets are being implemented in the clinic for tissues such as the cornea,^37^ myocardium,^38^ and esophagus.^39^ To form a cell sheet, cells can be cultured on thermally responsive tissue culture ware and removed via lowering the incubation temperature.^40,41^ A Common methodfor cell sheet culturing includes combining sheets together. For instance, Takahashi et al. expanded and combined three chondrocyte sheets and cultured them together for an additional 7 days to create the layered sheet used for implantation.^42^ Extensive *in vivo* work assessing the efficacy of chondrocyte cell sheets has been conducted in partial cartilage defect rabbit models,^43^ osteochondral defects in rats,^44^ rabbits,^45,46^ and minipigs.^47^ Furthermore, investigations regarding performed tumorigenicity and the monitoring of genetic mutations and abnormal chromosome emergence^48^ have allowed for the application of cell sheet technology in clinical trials in Japan.^49^

In the clinical aspect, a study conducted by Sato et al. utilized conventional surgical intervention for the treatment of knee OA which was followed up with autologous chondrocyte sheet transplantation to promote cartilage repair. The study was conducted using an eight-patient cohort and the success of the therapy was monitored 12 moths post operation using arthroscopic biopsies. Patient biopsies underwent histological analysis and cell sheets were evaluated using gene expression analysis to predict their clinical efficacy.^50^ MRI images were taken 36 months post operation. Images indicated that there was cartilage regeneration in defected areas and knee alignment was maintained. Results from this study support the idea that autologous chondrocyte sheets have the potential to support hyaline-like cartilage regeneration in human knee defects.^50^

Further advances in cell sheet engineering have begun to investigate different chondrocyte cell sources aside from autologous chondrocytes. A study conducted by Takao et al. used human iPSC-derived expandable limb-bud mesenchymal cells for chondrocyte sheet fabrication.^51^ Prior work from these investigators demonstrated the successful differentiation of human iPSCs into the induced limb-bud cells used in their current work.^51,52^ This study found that expandable limb-bud mesenchymal cells were functional and could produce engraftable chondrocyte sheets. The utilization of stem cells for the generation of cartilage has become more prevalent at an attempt to overcome the limitations of autologous chondrocyte expansion. In the next section, we will further expand upon the different stem cell therapies for cartilage repair.

### Stem cell therapies

Stem cells are a common and abundant cell type for cell therapies in a multitude of organs and pathologies. In this section, we will discuss the use of stem cell therapies for cartilage defects and investigate stem cell therapies in OA. Aside from the patient’s native bone marrow-derived stem cells, other stem cell sources are being studied as a cell therapy for cartilage regeneration. Of note, Table 1 describes the different cell sources and their advantages and limitations for cartilage regeneration. In this section, we will discuss mesenchymal stromal cells (MSCs), the MSC secretome, and iPSCs.

**Table 1.** Cell types for cartilage regeneration. A brief list of cell types, subtypes, sources and the advantages and disadvantages of each.

Cell Types	Cell Subtypes	Sources	Advantages	Limitations
Autologous Chondrocytes	None	Patient cartilage^53^	No immunogenicity^29^ Easeof procurement^29^	Additional surgery^53, 54^ Costly^53, 54^ Complications regarding phenotype^16, 55^
Stem Cells	Embryonic Stem Cells (ESCs)	Human embryos^56^	Pluripotency^57^ Unlimitedcell source^58^	Ethical concerns^55, 59^ Teratoma formation^56^ immunogenicity^55, 56^
	Induced Pluripotent Stem Cells (iPSCs)	Blood^60, 61^ fibroblasts^56^	Pluripotency^57^ Unlimited cell source^55, 58^ No immunogenicity^57^	In preclinical studies^59^ Teratoma formation^57^ Unsure of complete hyaline nature of cartilage^29^
	Mesenchymal Stromal Cell (MSCs)	Bone marrow^62^ (BMSCs) Adipose tissue^62^ (ADSCs) Umbilical Cord^63^	Unlimited cell source^16^ No immunogenicity^53^ Immunomodulation for anti-inflammation^63^	Variability of cell expansion due to aging^64^ Phenotypic alterations during differentiation^55, 64^ Intraarticular injection concern of “washout”^53, 62^
Synthetic Cells	None	Synthetically engineered chondrocytes^65^ iPSCs^66^	Personalized approaches via genetic manipulation^67^ No immunogenicity^68^ Unlimited cell source^68^	Limited preclinical work has been conducted^69^ Costly^70^ Ethical concerns regarding the use of lentiviruses^71^

#### Mesenchymal stromal cells

MSCs can be easily derived from the umbilical cord, bone marrow, adipose tissues, and have the ability to self-renew and differentiate into a multitude of tissues.^16, 29, 72^ More importantly, MSCs have the multipotent potential to differentiate into both chondrocytes and bone allowing them to be used for chondral and osteochondral defects.^12,29^ Specifically, bone marrow derived MSCs (BM-MSCs) show potential for articular cartilage regeneration when paired with trophic factors such as transforming growth factor-beta 3 (TGF- β3).^73^ Bioactive proteins from these MSCs have been shown to decrease T-cell activity in the damaged tissue and can act as a vehicle for therapeutic effects.^29,74^ MSCs contain hypoimmunogenic and immunosuppressive properties allowing for allogenic MSCs to be used in human tissue without HLA matching.^75^ Other studies have found that MSCs aid chondrocytes with phenotype maintenance during *in vitro* expansion,^76,77^ suggesting that the co-culture of MSCs and chondrocytes could be a new approach for MACI. Clinical studies using the intra-articular injection of bone marrow derived MSCs in OA patients have shown cartilage improvement and no serious effects.^78–80^ There have been questions regarding effective cell density needed for a therapeutic effect. A meta-analysis conducted by Muthu et. Al investigated the current randomized controlled trials available in literature to assess the most effective range of MSCs used for the treatment of OA. Here in, 14 studies including 564 patients were analysed and it was determined that the range of cell dose with the most therapeutic effect was 5-7×10^7^ MSCs.^81^ The lack of standardization among clinical trials contributes to the varying results of therapeutic efficacy of stem cell treatment for OA. It has been recommended that dose-escalation clinical trials be conducted to better standardize the use of stem cells in the treatment of OA.^81^

Adipose tissue-derived MSCs also known as adipose-derived stem cells (ADSCs) have become increasingly investigated due to their ease of procurement and therapeutic effect. The intra-articular injection of ADSCs have been tested in clinical trials for OA as well and showed similar results to the bone marrow MSCs.^82,83^ These cells have demonstrated increased interest as a potential stem cell source for cartilage regeneration due to their ease of accessibility, increased proliferative potential, and anti-inflammatory traits.^22^ The isolation of ADSCs is minimally invasive and cells are easily isolated from subcutaneous fat.^84^ Additionally, ADSCs have low immunogenic reactivity due to the limited expression of immunogenic antigens such as CD40, CD40L, CD80, and CD86.^85^ A study conducted by Lin et al. investigated the application of ADSCs in calcium-alginate hydrogel spheres and applied to a monosodium iodoacetate (MIA)-induced OA *in vivo* model in rats. Results from this study demonstrated increased walking performance of 3D- spheroid-culture ADSC treated rats compared to the 2D-culture ADSC treatment group. It was reported that the OA score was significantly reduced in the spheroid group as well.^84^ A clinical study using the intra-articular injection of autologous ADSCs demonstrated an improvement in pain, function and mobility in patients with severe knee OA.^83^ A meta-analysis comparing ADSCs and BM-MSCs found that ADSCs had a statistically significant and consistent improvement of outcome measurements compared to BM-MSCs. It was noted by this study that adipose tissue was a superior source of MSCs compared to bone marrow for the treatment of knee OA.^86^ Other meta-analysis have suggested the same idea; ADSCs have had better functional outcomes for the treatment of knee OA.^87^ However, further studies should be conducted to validate these findings.

The differentiation of ADSCs into chondrocytes has also been conducted. Studies have demonstrated a 21–28 day differentiation period and involves the incorporation of common chondrogenic growth factors such as TGF-β1 and TGF- β3, bone morphogenic proteins (BMPs), specifically BMP-4, sex determining region Y box 9 (SOX9), and basic fibroblast growth factor.^88^ Other studies suggest using the more heterogeneous adipose-derived stromal vascular fraction (AD-SVF) which is mechanically and/or enzymatically isolated from fat tissue. The AD-SVF poses a unique therapeutic potential and has been compared to microfracture in the sense that it consists of a plethora of cell types and paracrine factors that have been shown to stimulate endogenous regenerative pathways.^89^ In regards to OA related research, there have been numerous clinical trials utilizing AD-SVF as an OA therapy.^90^

On the other hand, the treatment methods for OA using MSCs are hindered by phenotypic challenges.^91^ While still an abundant cell source, the procurement of a high cell yield from MSCs is a concern due to phenotypic changes of chondrocytes during long culture times.^64^ Furthermore, the replication of hyaline cartilage producing chondrocytes from MSCs has been a challenge,^64^ and MSC differentiation *in vivo* is also a concern.^12^ Further concerns about donor age and cell passage expansion pose a great limitation to autologous stem cell procurement, necessitating the employment allogenic procedures.^4^

While MSCs possess the potential as a cell source for cartilage regeneration, they also secrete paracrine factors such as cytokines, chemokines, and extracellular vesicles that are thought to promote healing and regeneration as well. Therefore, the MSC secretome has gained interest as a potential cell-free therapy, not only for cartilage regeneration but in the cardiac field too.^92,93^ Extensive proteomic studies have shown that MSCs secrete 50–100 nm extracellular vesicles, namely exosomes, that provide a therapeutic effect to damaged tissues.^94,95^ A study conducted by Zhang et al. isolated human embryonic MSC-derived exosomes and performed weekly intra-articular injections to osteochondral defects in rats. Results depicted that after 12 weeks of exosome treatment defects had complete restoration of cartilage and subchondral bone with hyaline-like features compared to the fibrous tissues formed in the PBS controls.^93^

A different study by Zhu et al. investigated the difference between exosomes isolated from synovial membrane derived MSCs and iPSC-derived MSCs for the treatment of OA. In this work, they isolated exosomes from the respective cell sources and used intra-articular injection to study the exosomal effects in a collagenase-induced OA mouse model. Both cell sources produced a regenerative outcome. However, the iPSC-derived MSC exosomes demonstrated a stronger effect based on macroscopic, histological, and immunohistochemical analysis.^96^ Lastly, a study conducted by Cosenza et al. investigated a combination of exosomes and microparticles. Microparticles express markers from the parental cells but are derived from cell membrane budding and still contain proteins, lipids, and nucleic acids.^97,98^ In this study, murine BM-MSCs were isolated and expanded. Microparticles and exosomes were isolated from the MSCs, and a collagenase-induced OA model was used in mice. The *in vitro* portion of this study investigated the role of these particles on chondrocyte homeostasis and macrophage polarization toward an anti-inflammatory phenotype. It was determined that the microparticles and exosomes were determined to promote a chondroprotective effect in the OA mice.^98^ Collectively, these studies suggest that the MSC secretome has the potential to promote cartilage regeneration *in vivo*, however, more work should be conducted to fully understand the mechanism in which the MSC secretome is able to promote regeneration prior to use in the clinic.^99^

#### Induced pluripotent stem cells

Similar to the pluripotent nature of embryonic stem cells, iPSCs are generated from somatic cells that undergo a de-differentiation process, first demonstrated by Shinya Yamanaka.^100^ Sources for iPSCs consist of the blood^60, 61^ and fibroblasts^68^ in which isolated cells are lenti-virally altered to express common embryonic markers such as octamer-binding transcription factor 4 (Oct4), SRY-box 2 (Sox2), Kruppel-like factor 4 (Klf4), and c-myc.^55^ Further analysis of iPSCs have allowed for the creation of the iPSC library. This “library” allows for the procurement of iPSCs from multiple donor types allowing for the creation of iPSCs from homozygous donors matching a specific HLA, reducing the potential for immune rejection.^68^ As previously stated, iPSCs are pluripotent compared to the multipotency of MSCs, meaning that iPSCs can differentiate into any of the primary germ layers: ectoderm, mesoderm, and endoderm.^71,100^ These cells possess high self-renewal potential making them an ideal cell source for hyaline cartilage.^64,101^ Since cartilage tissue falls within the mesodermal layer, iPSCs can first undergo differentiation into mesenchymal progenitor cells (iMPCs) and then be differentiated into cartilage.^71,101^

There are limitations to the use of iPSCs/iMPCs: heterogenous cell populations following de-differentiation can lead to possible teratoma formation, high costs, and the challenge of replicating the hyaline cartilage *in vitro*.^29,102^ Specifically, the cell yield from iPSCs can be variable and the expansion/differentiation time can be extensive and costly.^103^ Additionally, there are medical ethical concerns faced by iPSCs/iMPCs regarding the methods used to obtain the de-differentiation. Viral vectors such as retroviruses and lentiviruses alter the genome of the original cell, causing concern for the ethical use of these genetically altered cells in the clinic. Infiltrating viruses effect the cell’s genome at random and can disrupt the function of the integrated genes and/or create genomic instability resulting in tumor formation.^104^ To avoid concerns regarding viral interference to the cell genome, a study conducted by Stadtfeld et al. was able to successfully create iPSCs without the use of viral integration through adenoviral reprogamming.^105,106^ Other methods consist of episomal vectors,^107,108^ Sendai viral vectors,^109^ expression plasmids,^110^ and different mRNA’s.^111,112^ The primary limitation of these different methods is the transfection rate resulting in a heterogenous cell population. Further investigation of complete reprogramming methods and additional aspects should be considered for a homogenous cell line of iPSCs.^104,113^

Despite the limitations mentioned, numerous studies have begun to investigate the differentiation of iPSCs into to cartilage in both *in vitro* and *in vivo* applications. There has yet to be a clearly established protocol that differentiates iPSCs into hyaline-like cartilage producing chondrocytes.^114,115^ As mentioned before, it is common for a two-step differentiation process to occur when producing iPSC derived chondrocytes. First, the iPSCs undergo a mesodermal differentiation into iMPCs and from there a subsequent process occurs to induce chondrogenesis. The induction of the iMPCs into chondrocytes can be accomplished through growth factors such as TGF- β and BMPs.^101^ Fibroblast growth factors (FGFs) are also linked to chondrogenesis. FGF-18 anabolically regulates cartilage tissue, and FGF-2 plays a role in the maintenance of cartilage homeostasis.^116^ As mentioned previously, TGF- β has been widely used and is well known for its ability to induce chondrogenesis in stem cells.^104,117^ The TGF- β family is commonly expressed in cartilage tissue and has been closely studied with MSCs to promote chondrogenic differentiation *in vitro*. ^118,119^ In combination with other growth factors, TGF- β and BMP-2, the differentiation of collagen II producing chondrocytes has been successful.^118^ Other studies have used platelet-derived growth factor -BB (PDGF-BB) in combination with TGF- β3 to begin the chondrogenic differentiation.^120^ These findings indicate that the use of stem cells such as iMPCs can be used to successfully create type II producing chondrocytes *in vitro.*

Common methods for chondrogenesis in iPSCs is the creation of embryoid bodies. Embryoid bodies are aggregates of pluripotent stem cells that can differentiate into the three germ layers.^104,121^ Embryoid bodies can undergo chondrogenesis. Once chondrogenesis is complete, they can be injected or implanted into the knee cartilage defect as demonstrated by Zhu et al.^103^ In this study OA was induced in rats with MIA injection. After 1 week of MIA administration, 500 uL of differentiated iPSCs were injected into the knee at a cell suspension density of 1 × 10^6^ cells/mL. This study used a three-step differentiation method of iPSCs, first into embryoid bodies, pre-induction of embryoid bodies in suspension culture and then the outgrowth of the cells on tissue culture dishes. Chondrogenic induction medium included ascorbic acid 2-phosphate, L-proline, dexamethasone, and TGF- β1.^103^ Other studies have used micromass or pellet culture to determine the efficacy of iPSC derived chondrocyte production of articular cartilage.^61,122^ In a study performed by Lee et al. mesoderm-derived cells and neural-crest derived cells were pelleted and underwent 21 days of chondrogenesis then implanted into mice for 30 days for ectopic hyaline cartilage generation. These pellets were also observed in rats to repair osteochondral defects. It was demonstrated that the neural crest derived chondrocytes showed phenotypic similarities consistent with hyaline cartilage chondrocytes during osteochondral defect regeneration.^120^

A unique approach to iPSC chondrogenesis was the use of mechanical stimulation. In the native knee joint chondrocytes often experience mechanical load which induces various stress and strains on the cartilage. A study conducted by Limraksasin et al. investigated mechanical shaking of 3D mouse iPSC constructs that were undergoing chondrogenic induction. Constructs were differentiated for 3 days in static culture and cultured then for 17 days on a see-saw shaker. Results from this study indicated that mechanical stimulation significantly promoted cell aggregation and showed higher expression of chondrogenic-related genes compared to static culture counterparts. It was also determined that the shaken group demonstrated activation of the TGF- β signaling pathway which plays an essential role in cartilage development.^123^ A different study by Aisenbrey et al. incorporated dynamic loading and growth factors to promote chondrogenic differentiation in iMPCs encapsulated in a hydrogel.^114^ iMPCs were encapsulated in a poly(ethyleneglycol), chondroitin sulfate, and adhesion peptide RGD hydrogel and TGF- β3 and BMP-2 were used to promote chondrogenesis. Dynamic compression occurred for three weeks with hydrogels cultured in custom bioreactors which were “subjected to intermittent unconfined dynamic compressive strains applied at 5% peak to peak strain (2.5% amplitude strain) at 1 Hz in a sinusoidal waveform one hour daily and with 23 hours of rest under a tare strain of < 0.1%.”^114^ Results indicated that dynamic compression in hydrogels supported chondrogenesis with limited hypertrophic gene expression only when TGF- β3 was administered. In groups lacking TGF- β3, there was an upregulation of collagen type X, which is indicative of a hypertrophic state. It was noted that the iMPCs indicated a transition into a hypertrophic phenotype prior to encapsulation, and following encapsulation became rapidly more hypertrophic.^114^ Moreover, the study from Limraksasin et al. and Aisenbrey et al. demonstrate the importance of mechanical stimulation for chondrogenesis and provide insight on its role in the promotion of hyaline-like cartilage producing chondrocytes derived from iMPCs.

It has been thought that iPSCs have epigenetic memory and despite their de-differentiation can still retain a memory of their native state. This memory is thought to be related to DNA methylation and histone modifications at lineage-specific genes.^124^ A recent study conducted by Khan et al. investigated articular chondrocytes as the cell source of iPSCs and compared healthy (AC-iPSCs) and OA-derived (OA-iPSCs). The goal of this study was to assess the chondrogenic potential of healthy and diseased chondrocyte-derived iPSCs, and whether the initial pathological state greatly alters their differentiation capacity. Results determined that the AC-iPSCs had increased chondrogenic potential compared to OA-iPSCs. The OA-iPSCs did retain alterations in metabolic factor production and epigenetics that correlated with a diseased chondrocyte state. Here in, it was determined that epigenetic memory has the potential to influence regenerative capacity and chondrogenic commitment.^124^ The genomic analysis of iPSC chondrogenesis has been investigated by Wu et al. in which single cell transcriptomic analysis of human iPSCs was employed throughout the differentiation of hiPSCs to map the genomic changes that occur during the differentiation process. This study determined that the *WNT* and *melanocyte inducing transcription factor* (*MITF*) genes were related to off target differentiation into neural cells and melanocytes during the chondrogenic differentiation process.^115^ It was found that the inhibition of WNT also prevents chondrocyte hypertrophy and limits the differentiation of off target cell populations during iPSC chondrogenesis.^115^

Tissue engineers are working toward developing methods for 3D bioprinting scaffold-free cartilage constructs for cartilage regeneration.^120^ A study conducted by Nakamura et al. used iPSC derived neural crest cells and induced them into an MSC-like state. The cells were differentiated and shaped into spheroids that were used in the bioprinting. This study used the bioprinter to construct different shapes such as tubes, L-shape, and a surface shape designed to mimic the shape of the articular surface.^120^ While these methods have yet to be implemented in the clinic, further research is being conducted with iPSCs for cartilage defect regeneration.^120^ iPSCs can be applied to personalized medicine as they can be derived from the patient themselves limiting immune response.^55^ However, understanding the complexities associated with iPSC differentiation into chondrocytes must be done prior to implementation in the clinical setting to avoid ethical concerns.

Understanding the multiple aspects of OA and preexisting joint disease is necessary for patient-based therapy development. Moreover, understanding the cause of the patient’s OA such as mechanical joint failure, inflammatory stresses, and metabolic stress can elucidate the best method of treatment for these patients. The lack of understanding in the regenerative cascade of cartilage regeneration has led to inconsistencies in treatment approaches and therefore inconsistent outcomes.^99^ It has been suggested in order to mitigate these outcomes, future trials should test specific hypotheses and focus on the mechanistic understanding of cartilage biology and regeneration in order to provide valuable information for the development of the treatments.^99^

## Future directions: potential of synthetic biology for cartilage regeneration

**Figure 3.**
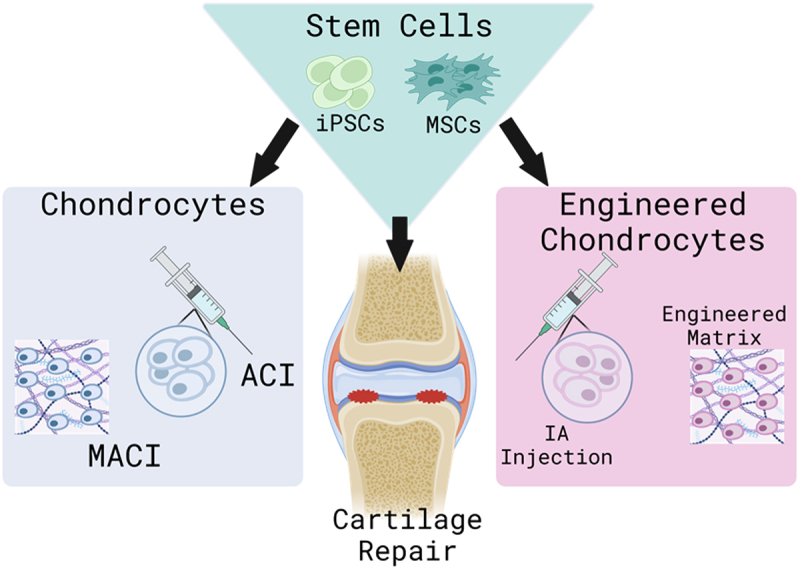
The multiple cell therapies for cartilage repair. This schematic depicts the multiple cell types and uses for cartilage defect repair. Here, we begin with autologous chondrocytes as seen in ACI and MACI. Then we move to stem cells, primarily MSCs and iPscs, which can be differentiated into chondrocytes and used in IA injection and matrices. The future of cell therapy focuses primarily with synthetically engineered chondrocytes or stem cell-derived chondrocytes for cartilage regeneration.

In other disease states such as cancer, cells have been actively engineered using synthetic biology to target specific receptors. For instance, chimeric antigen receptor T-cells (CART-cells) have been engineered to target tumor associated antigen receptors.^125,126^ In the case of the aging population with relation to OA, there is i) a lack of chondrocyte regenerative potential^127^ and ii) the common onset of cellular cell cycle arrest known as senescence, which induces a chronic low-grade state of inflammation through the onset of the senescence associated secretory phenotype (SASP).^128^ It is possible to employ synthetic applications to increase chondrocyte regenerative potential and create therapeutic processes through synthetic genetic networks essentially creating an “engineered chondrocyte” as shown in Figure 3.

Studies exist focusing on synthetic gene circuits to engineer cells to respond to intracellular signals for the creation of cell-secreted therapeutics. For example in a study conducted by Nims et al. used the activation of transient receptor potential vanilloid receptor 4 (TRPV4) to drive synthetic gene circuits in chondrocytes resulting in the production of anti-inflammatory molecule interleukin receptor antagonist (IL-1Ra).^65^ Moreover, this study provides insight on the efficacy of synthetically engineered chondrocytes and their potential as a source for cell therapy in OA. Another study conducted by Wu et al. investigated the regulatory pathways associated with iPSC chondrogenesis and determined that inhibiting WNT pathway signaling in human iPSCs might reduce the heterogeneity of iPSC chondrogenesis. Using a WNT inhibitor and bioinformatics, they were able to determine that WNT inhibition does result in better iPSC chondrogenesis.^115^ The unique features of synthetic biology can be extended into multiple cell types such as MSCs. Huynh et al. genetically altered MSCs to optimize chondrogenesis and osteogenesis using short hair pin RNA and lentiviral vectors. In this study, they demonstrate the ability to engineer cells to optimize differentiation potential in a biomaterial-based scaffold.^129^ These studies demonstrate the potential of synthetic biology for cell therapy and tissue engineering.

Synthetic biology can also be used to address the concerns associated with off-target differentiation of iPSCs. One of the primary limitations of iPSCs was the lack of a standardized protocol for chondrogenesis. In order to address this limitation, a study by Adkar et al. used CRISPR-Cas9 genome editing to create a reporter for the stepwise analysis of iPSC chondrogenesis. Through the creation of a *collagen type II alpha 1 chain- green fluorescent protein* (*COL2A1-GFP)* knock-in reporter, the chondrogenesis of three different human iPSC cell lines could be tracked throughout the differentiation process. The goal of this study was to observe iPSC chondrogenesis with hopes of understanding different methods to create a homogeneous cartilage matrix devoid of off target cell populations. Because collagen type II is the primary collagen type associated with the production of hyaline cartilage, the use of the *COL2A1-GFP* reporter allows for the precise monitoring of *COL2A1* expression throughout the chondrogenesis process and aids in the purification process of chondrocytes during iPSC chondrogenesis.^130^

A different study used CRISPR/Cas9 to delete the *collagen type X alpha 1 chain* gene (*COL10A1*) in iPSCs to investigate the role of *COL10A1* in chondrocytes. Here, Kamakura et al. established iPSCs with heterozygous (*COL10A1* ^*+/−*^) or homozygous (*COL10A1* ^*−/−*^) deletions of *COL10A1*. This study focused on the growth plate and hypertrophic chondrocytes. Chondrocytes undergoing hypertrophy are thought to be a source of osteogenic cells.^131,132^ Collagen type X is a marker of chondrocyte hypertrophy. The knockout of *COL10A1* in human iPSCs demonstrated that the loss of function of COL X does not affect hypertrophic differentiation of chondrocytes or endochondral bone formation, but may contribute to the differentiation process of iPSC derived chondrocytes.^133^ While this study focused on the growth plate rather than hyaline cartilage, it still demonstrates the use of synthetic approaches to promote chondrogenesis in stem cells. Here in, we can utilize synthetic biology approaches to engineer chondrocytes and stem cells through CRISPR/CAS9 for increased cartilage regeneration and repair.^67,69^ Harnessing the inherent potential of the cell to optimize the regeneration of a specified tissue might be the forefront of tissue engineering.

A common concern regarding iPSCs was the medical ethical considerations of using lentiviruses and retroviruses and potential alterations to the cell’s genome. It is understood that the use to synthetic biology in the clinical setting could also raise the same ethical concerns. However, extensive studies must occur prior to the application of synthetic cells in the clinical setting, as well as the approval of all cell-based therapeutics in general. For example, CART-cells are genetically engineered cells that have shown success in treating cancers. The employment of synthetic approaches might face some regulatory challenges before their approval in the clinical setting, but with extensive studies demonstrating the safety and efficacy of synthetic cells in pre-clinical and clinical trials there is still hope that these approaches might make reach FDA approval. With any medical device development there will be challenges and setbacks, but the evolution of the scientific field necessitates growth and development.

## Conclusion

In this review, we discussed the current clinical approaches for cartilage regeneration, primarily in patients under the age of 45. ACI and MACI have demonstrated success for cartilage defects but are limited in the breadth of patients that are eligible for this procedure. Additionally, there are not any current DMOADs that have reached FDA approval and stem cell therapies for patients with OA are still in early-stage clinical trials. Understanding the effects of the preexisting joint pathology is necessary to develop therapies. Stem cells such as MSCs and iPSCs are an unlimited cell source for cartilage regeneration but come with their own limitations such as cost, ethical and phenotypic challenges. Employing synthetic biology techniques for enhanced cartilage regeneration might aid in the lack of promising cell therapies for OA.
